# Porphyra-334 Isolated from the Marine Algae *Bangia atropurpurea*: Conformational Performance for Energy Conversion

**DOI:** 10.3390/md12094732

**Published:** 2014-09-03

**Authors:** Li-Fan Chuang, Hong-Nong Chou, Ping-Jyun Sung

**Affiliations:** 1 Institute of Fisheries Science, National Taiwan University, No. 1, Sec. 4, Roosevelt Rd., Da-An district, Taipei 106, Taiwan; E-Mail: unijohn@ntu.edu.tw; 2 Institute of Marine Biotechnology, National Dong Hwa University, Pingtung 944, Taiwan; E-Mail: pjsung@nmmba.gov.tw

**Keywords:** porphyra-334, p*K*_a_, titration, conformation

## Abstract

Prophyra-334 (p-334) may play a role of energy transfer under an uncertain mechanism, and we speculate the possible model. Via 1D and 2D NMR experiments, it was simulated the correlation between dissociation and conformation of p-334. Intramolecular interactions were observed based on a series of changes in the 1H and 13C chemical shifts. Nuclear Overhauser effect spectroscopy experiments and molecular models in various pD conditions indicated the p-334 molecular dissociation process status. In addition, we also used Chem3D software to find the most possible molecular conformation. The relationship between the structural status and energy conversion is explained. Those are the primary results. More researches on it are highly expected in the future.

## 1. Introduction

**Figure 1:**
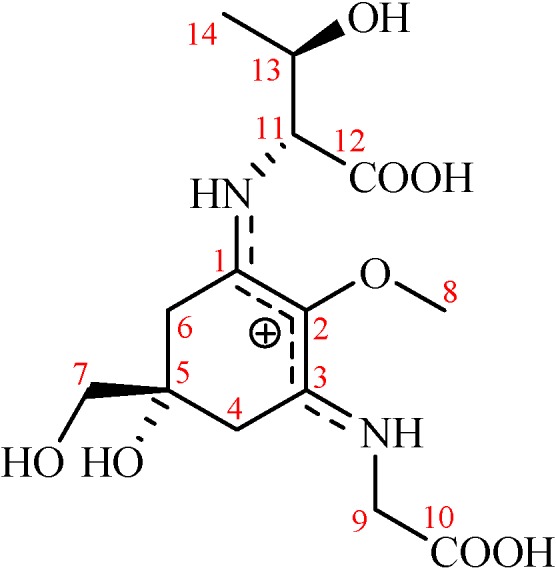
The molecular structure of porphyra-334.

Porphyra-334 (Figure 1; p-334; λ_max_ = 334 nm; ε = 42,300 M^−1^·cm^−1^) belongs to the class of mycosporine-like amino acids (MAAs) that is a common constituent of algae and aquatic organisms [1]. It has been observed in high concentrations in some algae, particularly *Porphyra* spp. and *Bangia atropurpurea* [2,3]. This compound has been proved to be an activator of cell proliferation [4] and an antioxidant [5]; moreover, it is a UV protector [6] and is currently being explored regarding potential use in the cosmetics industry as a sunscreen agent [7]. In addition, p-334 can absorb light energy, and more than 90% of the excitation energy can be transferred to heat, which is released to the surrounding molecules; thus, p-334 can be considered an energy-converting substance [8,9].

In previous studies, p-334 was stable in a medium after 25 h in environments in which the pH was between 2 and 11 and the temperature was below 40 °C [10]. These results suggested that p-334 is stable and can be passed safely through the stomach and accumulated in organisms. P-334 is monomeric in a D_2_O solution, and it has been suggested to be the imino N-protonated form of p-334 based on the results of *ab initio* ^13^C-NMR chemical shift calculation and NMR Predict software [11]. These results indicate that p-334 should carry a positive charge in a solution and that energy conversion involves electron transfer and the structural status. However, this zwitterion compound may exhibit a negative charge or no charge in organisms, prompting us to study whether one or two protons are ionized from these carboxylic acid groups and to clarify the correlation between the structure and energy conversion. This experiment was conducted to determine p*K*_a_ values by using various NMR chemical shifts. In addition, p-334 is an efficient proton sponge that is expected to have a relationship with intramolecular interactions [11]. The structural conformations of the ionic and neutral forms were determined using nuclear Overhauser effect spectroscopy (NOESY). Furthermore, the p-334 effect relative to cell proliferation is addressed.

## 2. Results and Discussion

According to the progress of extract filtration, p-334 was easily extracted from the algae *B. atropurpurea*, indicating that MAAs exist between the cell wall and the membrane. This is the first time this observation has been reported, and it may be related to the energy conversion of UV light into heat considered the growth environment. This topic will be studied further in the future. After p-334 was eluted from the ion exchange column, the pH of the eluent was observed to be approximately 3.0; this value is close to the measured p*K*_a_ value from NMR.

**Figure 2:**
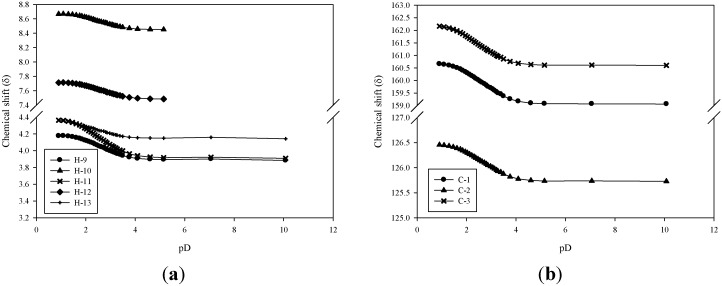
pD dependence of the (**a**) ^1^H chemical shift and (**b**) ^13^C chemical shift of p-334.

The results of titrations indicated that the hydrogen molecules around two of the carboxylic acid groups were affected, and chemical shifts changed before pD 4 (Figure 2). However, we calculated equivalents and demonstrated that only one proton ionised from a carboxylic acid. The p*K*_a_ was evaluated according to the equation p*K*_a_ = 0.929{pD + log ((δ–δ_n_)/(δ_i_–δ))} + 0.42, in which δ is the chemical shift at a given pD, and δ_n_ and δ_i_ indicate the intrinsic chemical shift values for the nonionic and ionic species, respectively [12,13]. The values calculated from Figure 2a were 2.95 ± 0.09 (H-9), 2.89 ± 0.10 (H-10), 2.91 ± 0.14 (H-11), and 2.96 ± 0.11(H-12), and the average was 2.92 ± 0.11. Figure 2b provides values of 2.94 ± 0.11 (C_(1)_), 2.96 ± 0.12 (C_(2)_), and 2.85 ± 0.12 (C_(3)_) for the p*K*_a_, and the average was 2.92 ± 0.12. This is the same p*K*_a_ calculated based on ^1^H and ^13^C NMR chemical shifts, indicating that the structure contained intramolecular interactions and that change occurred upon proton ionization.

**Figure 3:**
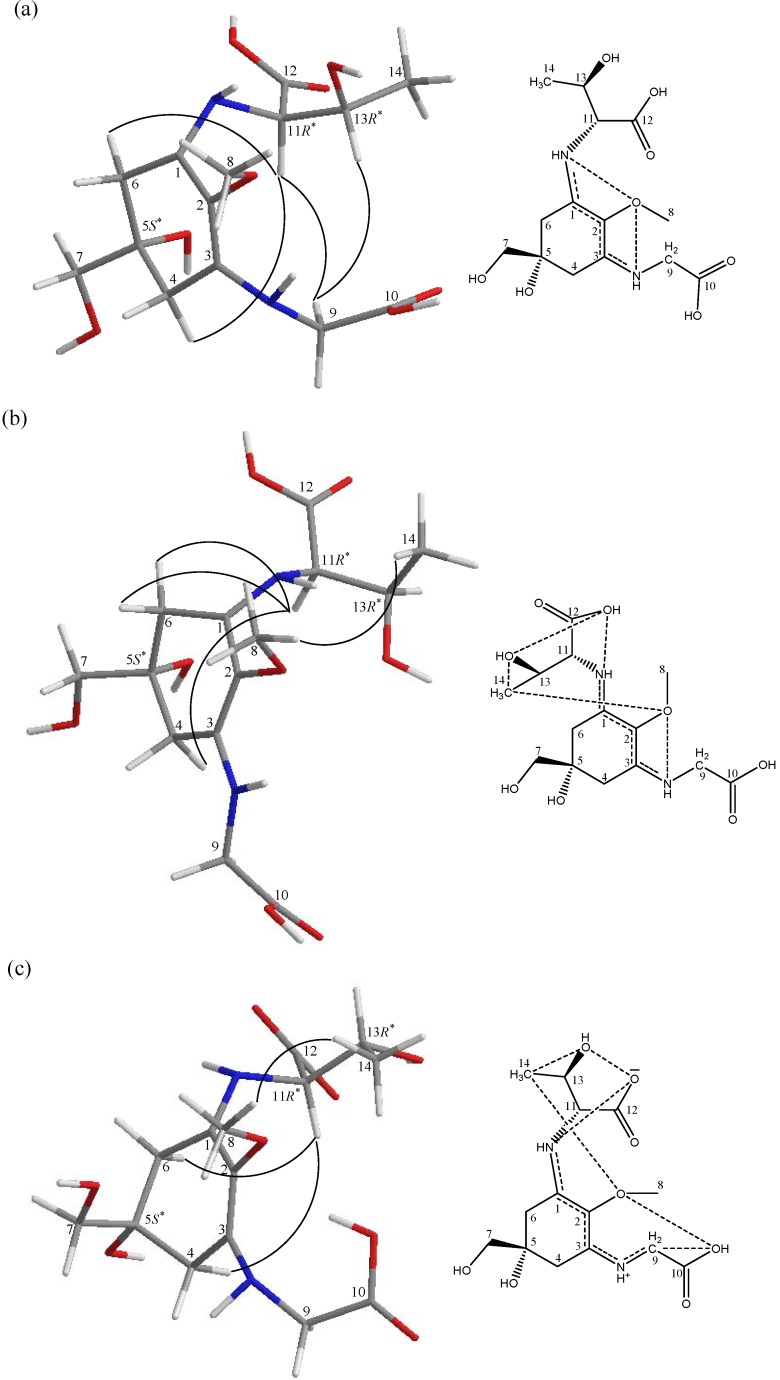
Selective NOESY correlations of p-334 and the possible process of conformational change as shown by molecular models under different pD conditions. The dashed lines in the chemical structures of the scheme represent possible paths of electron resonance. (**a**) p-334 dissolved in D_2_O and titrated by DCl (pD 1.0); (**b**) also titrated by DCl (pD 2.0); (**c**) no treatment (pD 3.5).

The NOESY experiments revealed that p-334 exhibited various conformations in several pD conditions (Figure 3). At pD 1.0, NOESY correlations between H-11 and H^α^-4, H^α^-6, and H_2_-9, as well as between H-13 and H_2_-9 were observed. However, at pD 2.0, NOE correlations were observed between H_3_-14 and H_3_-8, and between H-11 and both H^α^-4 and H_2_-6. At pD 3.5, H-11 was observed to engage in NOE interactions with only H^α^-4 and H^α^-6, and H_3_-14 interacted with H_3_-8. The intramolecular interactions increased the stability of the conformation [11]; for example, at pD 1.0, C_(1)_NH interacted with C_(2)_O and C_(13)_O, H-13 interacted with C_(2)_O, and H-11 interacted with C_(5)_O, preventing the NH of the threonine side chain from rotating freely. The NH of the glycine side chain also exhibited contact between C_(3)_NH to C_(10)_O and C_(2)_O (Table 1).

Figure 2a shows that the shifting distance of H-11 was two times greater than that of other types of hydrogen, suggesting that the change in electron density was two times greater than that of other types of hydrogen. Between pD 1.0 and pD 3.5, H-13 interacted with C_(2)_O initially, but then interacted with C_(12)_O. H_3_-14 exhibited a reversed process; therefore, it maintained the same electron density (Table 1). However, the hydrogen of C_(13)_OH interacted with C_(12)_O, causing the electron densities of H-13 and H-11 to increase. In addition, hydrogen ionization from C_(12)_OH caused the electron density of H-11 to increase twofold. Thus, the dissociated proton may have been located in the carboxylic acid group of the threonine side chain, and the hydrogen of C_(13)_OH may have interacted with H-12, producing a hydrogen signal from ^1^H-NMR. According to these findings, the hydrogen of C_(10)_OH may have interacted with C_(2)_O, engendering the increased electron densities of H-9 and H-10 simultaneously.

**Table 1:** The calculated distances below 3.00 Å between selected protons and oxygens by molecular models.

pD	1.0		2.0		3.5	
Proton	Oxygen	(Å)	Oxygen	(Å)	Oxygen	(Å)
C_(1)_N-H ^a^	C_(2)_O	2.21	C_(2)_O	2.20	C_(12)_O	1.64
	C_(13)_O	1.98	C_(13)_O	2.44	-	-
C_(3)_N-H ^b^	C_(2)_O	2.14	C_(2)_O	2.30	-	-
	C_(10)_O	2.09	C_(10)_O	2.58	-	-
C_(5)_O-H	C_(7)_O	2.15	C_(7)_O	2.11	-	-
H_2_-9	C_(10)_O	2.61	C_(10)_O	2.58	C_(10)_O	2.60
C_(10)_O-H	-	-	-	-	C_(2)_O	2.09
H-11	C_(5)_O	2.50	C_(5)_O	2.45	C_(12)_O	2.08
	C_(12)_O	2.70	C_(13)_O	2.55	C_(13)_O	2.61
H-13	C_(2)_O	2.64	C_(12)_O	2.77	C_(12)_O	2.44
	C_(13)_O	2.01	C_(13)_O	2.04	C_(13)_O	2.03
C_(13)_O-H	-	-	-	-	C_(12)_O	1.85
H_3_-14	C_(12)_O	2.57	C_(13)_O	2.64	C_(2)_O	2.61
	C_(13)_O	2.63	-	-	C_(13)_O	2.59

^a^ The NH group on the threonine side chain; ^b^ The NH group on the glycine side chain.

According to the results of these chemical shifts, C_(3)_ was determined to have a higher electron density than C_(1)_ and C_(2)_ do because C_(2)_O interacted with other types of hydrogen (Figure 2b). P-334 can be called a “proton sponge compound [11]”, because it can form strong intramolecular interactions. At pD 1.0, the cycles of the electronic orbitals were C_(8)_H_3_–O…H–N–C_(1)_…C_(2)_ and C_(8)_H_3_–O…H–N–C_(3)_…C_(2)_ (Figure 3a). However, at pD 2.0, some protons dissociated from C_(12)_OH, resulting in an increase in the electron density of the threonine side chain and causing the electron density around C_(12)_ to increase. The balance connection between the threonine side chain and the glycine side chain were broken. The electron density increased, and the mutual repulsion increased, thus causing the electron orbitals and conformation of p-334 to change. The mode of connection becomes C_(8)_H_3_–O…C_(14)_H_3_…C_(13)_O–H…C_(12)_O…H–N–C_(1)_…C_(2)_ and C_(8)_H_3_–O…H–N–C_(3)_…C_(2)_ (Figure 3b). We predict that, as more protons dissociate at pD 3.5, more C_(1)_NH interacts with C_(12)_O, and the distance between C_(1)_NH and C_(2)_O increases. Finally, interactions may form a cycle of C_(8)_H_3_–O…C_(14)_H_3_…C_(13)_O–H…C_(12)_O…H–N–C_(1)_…C_(2)_ and C_(8)_H_3_–O…C_(10)_H–O…C_(9)_H_2_…H–N–C_(3)_…C_(2)_ (Figure 3c). The widely electronic orbitals improve the stability and increase the proton affinity of the structure. These results also indicated that the electron density of C_(1)_ to C_(3)_ increased (Figure 2b). Zhang *et al.* (2005) [10] reported that the maximal absorption of p-334 exhibited a hypsochromic shift below pH 3. A hypsochromic shift on UV spectra indicates greater energy content; hence, we suggest that p-334 ionization is a key on energy transfer on-off.

Proton-coupled electron transfer (PCET) reactions are ubiquitous in energy conversion and storage reactions in biology [14]. The energy-conversion activity of P-334 might involve PCET reactions. The NAD(P)/NAD(P)H redox couple is a suitable model for explaining the role of p-334 in energy-conversion reactions [15,16]. However, energy cannot always do the conversion. If the structural conformation of p-334 in Figure 3c is the energy consumption status in organisms that will perform energy conversion, then the progress of p-334 ionization in Figure 3b should have as the appearance energy threshold [17,18,19]. Hence, Figure 3a is the energy storage status. The involvement of p-334 ionization in protonic systems might relative in light-driven proton pumps [20,21,22], explaining why MAAs are present in halophilic cyanobacteria [23,24] and fish eye tissues [25,26].

MAAs are present in the organs of marine organisms such as green sea urchin before spawning (*Strongylocentrotus droebachiensis*) [27,28]. MAAs accumulate in the ovaries during gastrulation, and larvae still contain MAAs; however, in the next stage of larval growth, the MAAs concentration decrease. In another urchin (*Sterechinus neumayeri*), the ovaries exhibit high MAAs concentrations, but the testes do not [29]. The ovaries of various types of fish, such as cod and common dab, contain approximately 4 mg·g^−1^ of mature eggs [30,31]. These observations support that MAAs act as energy-converting substances that facilitate cell proliferation and biological reproduction. The results of cell proliferation (0.25 µM) of 3T3 mouse fibroblast cells were accelerating growth by approximately 40%, that similar as Oyamada *et al.* studies [4]. Although the mechanism remains unclear, we believe that it is related to the aforementioned features of MAAs. Further research is required to confirm these observations. More details are available in Supplementary Information.

## 3. Experimental Section

### 3.1. Procedure for Purifying P-334

*B. atropurpurea* was purchased from the market, and was identified by algal professional, Prof. Chou. The following protocol was used to purify p-334: Extract filtration: Two steps occurred at nearly the same time that the “go-through” way was used. The extracts were filtered and the concentration was calculated according to the Beer-Lambert law. Absorbance was detected using a UV-VIS spectrophotometer. The concentration of p-334 in extracts was approximately 0.1%. Adsorption: The extraction was passed through an ion exchange chromatography column that contained 40 g of Dowex 50 W-X8 (200–400 mesh) gels by using a peristaltic pump, and the column was washed with H_2_O and eluted with 0.5 N HCl. The purity of p-334 was at least 50%. Eluate fractions containing p-334 were concentrated and dried. Purification: The p-334 was purified using a Cosmosil 140 C_18_-OPN chromatography column and eluted using isocratic 0.04% AcOH/H_2_O. According to high-performance liquid chromatography results [32], the purity was greater than 99%.

### 3.2. Properties of the P-334 Experiment

pD studies: The sample concentration of D_2_O was approximately 0.37 M. A glass electrode was employed in adjusting the pD from pD 0.9 to 3.4 by using diluted DCl and from pD 3.5 to 10.1 by using diluted NaOD. All measurements were conducted at 25 °C with ^1^H NMR (400 MHz) and ^13^C NMR (100 MHz) spectra which 32 and 256 scans, respectively. The ^1^H NMR chemical shifts were determined based on the signal of HOD peak. Computer processing was performed using the vnmr Varian software. Structural conformation studies: P-334 prepared at 0.05 M in D_2_O was used in the 2D NOESY experiments, in which the pD was adjusted to 1.0 and 2.0 were detected using a 400-MHz NMR spectrometer which recorded 32 scans at 25 °C. Proliferation assay: This assay was conducted according to the procedure described by Oyamada *et al.* [4]. Molecular models: These models were applied to calculate the minimal energy by using the Chem3D Ultra 9.0 software (PerkinElmer, Waltham, MA, USA). 

## 4. Conclusions

According to the data, proton ionization from the carboxylic acid group of the threonine side chain and the structural conformations of p-334 in three pD conditions revealed the progress of p-334 ionization. In addition, we suggest that p-334 ionization is a key on energy transfer on-off and that p-334 acts as an energy-converting substance that facilitates cell proliferation. This is a preliminary study and is necessary to acquire a specification of additional information that in the future.

## Supplementary Files

Supplementary File 1
