# Supplementary material for: Porphyra-334 Isolated from the Marine Algae Bangia atropurpurea: Conformational Performance for Energy Conversion
Source: Mar Drugs. 2014 Sep 3;12(9):4732–40. doi: 10.3390/md12094732 (PMC4178487; doi:10.3390/md12094732)
Supplement: Supplementary File 1 [file marinedrugs-12-04732-s001.pdf]

Supplementary Information

1. The "Go-Through" Way

(1) A schematic diagram of the "go-through" extraction and filtration system and a photograph of the collected sample are shown as follows (Figure S1):

Figure S1. A schematic diagram of the "go-through" extraction and filtration system and a photograph of the collected sample.

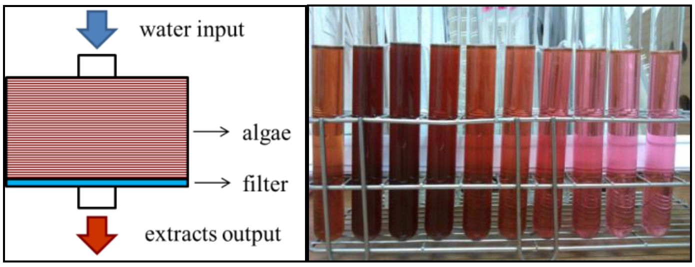

(2) The absorbance λ_{334} nm of every collected fraction was detected using a UV-VIS spectrophotometer. Figure S2 shows the results of separate operations conducted five times.

Figure S2. The absorbance λ_{334} nm of every collected fraction.

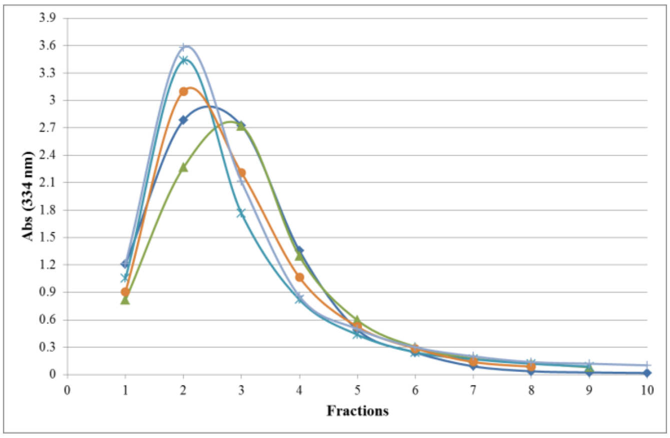

(3) The fractions of each extract were combined and the concentration of porphy-334 (p-334) in the extracts was approximately 0.1%. (*Bangia atropurpurea* contained a dry weight of approximately 10 mg g^{-1} of p-334)

2. Comparison of the Efficiency of Extraction

Table S1 shows a simple comparison of various extraction conditions. The p-334 content obtained using the go-through way did not differ from that obtained using other methods. Moreover, the volume extracted using the go-through way was lower than that extracted using other methods, indicating that p-334 was easy to extract.

Table S1. The different extraction conditions and the p-334 content.

The Extraction Conditions	Water Volume Used to Extraction(Dry Weight Per Water Volume)	Calculated Content by by Beer–Lambert Law Which Absorbance λ_{334} nm Detected by UV-VIS Spectrophotometer (mg)
r. t. for 30 min	1 g/30 mL	15.39
		17.17
r. t. for 10 min	1 g/25 mL	15.94
		14.07
go-through way	1 g/15 mL	16.37

3. 2D Nuclear Overhauser Effect Spectra

The pK_a of p-334 was 2.92 ±0.11, indicating that more than half of the p-334 molecules were ionised at pD 3.5. The nuclear Overhauser effect spectroscopy (NOESY) experiment was used to detect various conformational statuses of p-334 by observing all signals on the spectra. Three steps were implemented to retrieve data for the NOESY experiments. First, the data were compared with those of Klisch *et al.* (2007) [1] regarding NOE and NOESY experiments. Second, NOESY spectra data predicated dependent on the signal strength of spectra, and the ^{1}H-^{1}H correlation crossed together on a certain signal center of the NOESY spectra. Third, the results of the NOESY spectra were used to identify appropriate molecular models for calculating the minimal energy by employing the Chem3D Ultra 9.0 software. The 2D NOESY spectra and tabular data are shown as follows (Table S2).

Table S2. The ^{1}H-^{1}H NOESY correlation of p-334.

H	pD = 1.0	pD = 2.0	pD = 3.5	Klisch *et al.*, 2007 [1] NOE or NOESY
$4^α$	$4^β$, $6^α$, $6^β$, 7, 9, 11	$4^β$, $6^α$, $6^β$, 7, 9, 11	$4^β$, $6^α$, $6^β$, 7, 9, 11	$4^β$, $6^α$, $6^β$,7, 9, 11
$4^β$	$4^α$, $6^α$, $6^β$, 7, 9	$4^α$, $6^α$, $6^β$, 7, 9	$4^α$, $6^α$, $6^β$, 7, 9	$4^α$, $6^α$, $6^β$, 7, 9
$6^α$	$6^β$, $4^α$, $4^β$,11	$6^β$, $4^α$, $4^β$,7, 11	$6^β$, $4^α$, $4^β$,11	$6^β$, $4^α$, $4^β$, 7, 11
$6^β$	$6^α$, $4^α$, $4^β$, 7	$6^α$, $4^α$, $4^β$, 7, 11	$6^α$, $4^α$, $4^β$,7	$6^α$, $4^α$, $4^β$, 7, 11
7	$4^α$, $4^β$, $6^β$	$4^α$, $4^β$, $6^α$, $6^β$	$4^α$, $4^β$, $6^α$, $6^β$	$4^α$, $4^β$, $6^α$, $6^β$
8	-	14	14	14
9	$4^α$, $4^β$, 11, 13	$4^α$, $4^β$	$4^α$, $4^β$	$4^α$, $4^β$
11	$4^α$, $6^α$, 9, 14	$4^α$, $6^α$, $6^β$, 13, 14	$4^α$, $6^α$, 13, 14	$4^α$, $6^α$, $6^β$, 13, 14
13	9, 14	11, 14	11, 14	11, 14
14	11, 13	8, 11, 13	8, 11, 13	8, 11, 13

Figure S3. 2D NOESY Spectra (pD = 1.0).

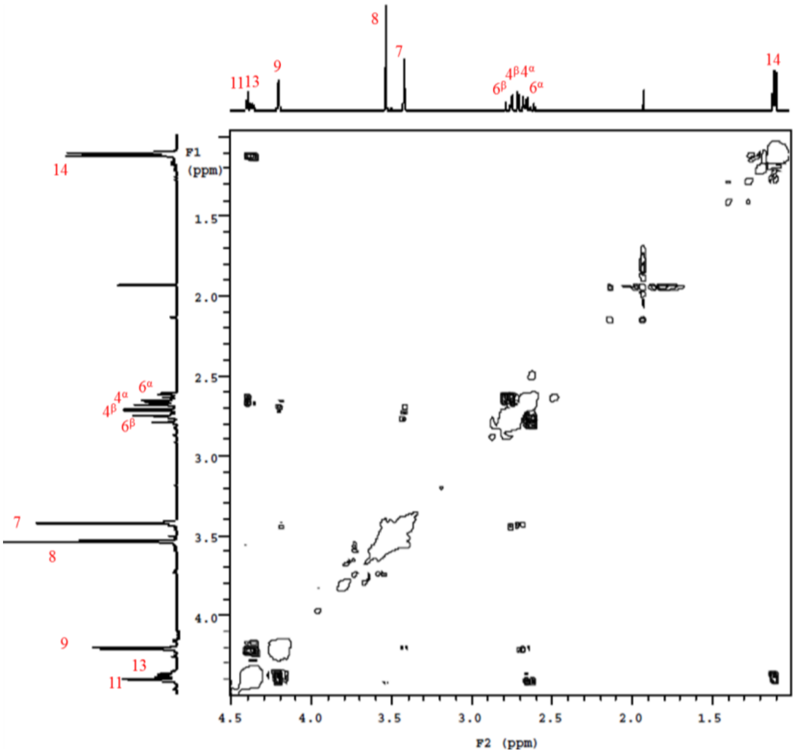

Figure S4. 2D NOESY Spectra (pD = 2.0).

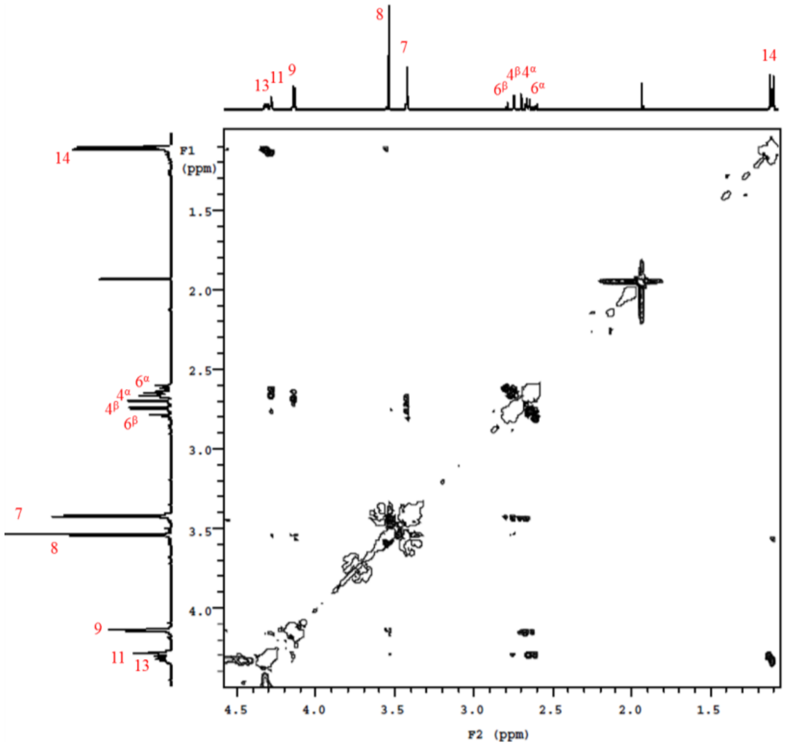

Figure S5. 2D NOESY Spectra (pD = 3.5) (un-titration).

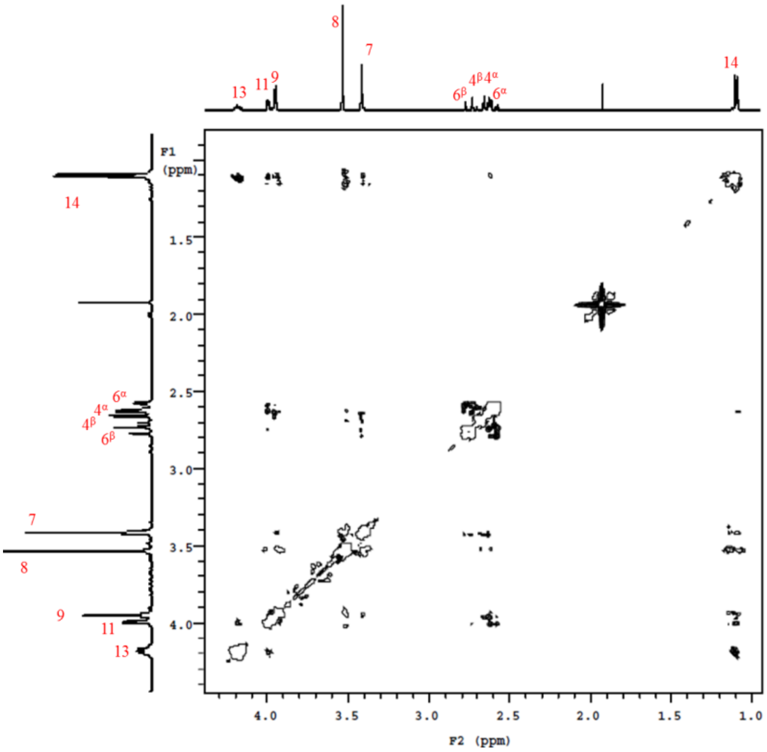

Reference

1. Klisch, M.; Richter, P.; Puchta, R.; Hader, D.P.; Bauer, W. The stereostructure of porphyra-334: An experimental and calculational NMR investigation. Evidence for an efficient "proton sponge". *Helv. Chim. Acta* **2007**, *90*, 488–511.

© 2014 by the authors; licensee MDPI, Basel, Switzerland. This article is an open access article distributed under the terms and conditions of the Creative Commons Attribution license (http://creativecommons.org/licenses/by/3.0/).
